# Protein–Protein Affinity Determination by Quantitative FRET Quenching

**DOI:** 10.1038/s41598-018-35535-9

**Published:** 2019-02-14

**Authors:** Ling Jiang, Zhehao Xiong, Yang Song, Yanrong Lu, Younan Chen, Jerome S. Schultz, Jun Li, Jiayu Liao

**Affiliations:** 1 Department of Bioengineering, University of California at Riverside, 900 University Avenue, Riverside, CA 92521 USA; 2 Department of Statistics, University of California at Riverside, 900 University Avenue, Riverside, CA 92521 USA; 3 Institute for Integrative Genome Biology, University of California at Riverside, 900 University Avenue, Riverside, CA 92521 USA; 4 Key Laboratory of Transplant Engineering and Immunology, NHFPC, West China Hospital, Sichuan University, ChengDu, Sichuan P.R. China; 5 Present Address: Admera Health Inc., Research and Development, 126 Corporate Blvd, Princeton, NJ 07080 USA; 6 Biochemistry and Molecular Biology Department, Heilongjiang University of Chinese Medicine, 24 Heping Road, Harbin, 150040 P.R. China; 7 Present Address: School Basic Medical Science, Heilongjiang University of Chinese Medicine, 24 Heping Road, Harbin, 150040 P.R. China

## Abstract

The molecular dissociation constant, *K*_*d*_, is a well-established parameter to quantitate the affinity of protein-protein or other molecular interactions. Recently, we reported the theoretical basis and experimental procedure for *K*_*d*_ determination using a quantitative FRET method. Here we report a new development of *K*_*d*_ determination by measuring the reduction in donor fluorescence due to acceptor quenching in FRET. A new method of *K*_*d*_ determination was developed from the quantitative measurement of donor fluorescence quenching. The estimated *K*_*d*_ values of SUMO1-Ubc9 interaction based on this method are in good agreement with those determined by other technologies, including FRET acceptor emission. Thus, the acceptor-quenched approach can be used as a complement to the previously developed acceptor excitation method. The new methodology has more general applications regardless whether the acceptor is an excitable fluorophore or a quencher. Thus, these developments provide a complete methodology for protein or other molecule interaction affinity determinations in solution.

## Introduction

Protein interaction affinity as characterized by dissociation constant *K*_*d*_ is one of most important parameters for protein interactions in various physiological and pathological processes. Recently, a new effort to take advantage of FRET technology to determine protein interaction affinity has emerged^1^. Traditionally, a ratiometric method (acceptor emission/donor emission) has been widely used in quantifying the FRET signal. However, the ratiometric method for FRET analysis is not accurate measurement of absolute FRET signal. For example, bleed-through excitation occurs when an acceptor is excited by the donor’s excitation wavelength. Also, crosstalk in emission detection occurs when the emission of a donor contributes to the signal at the wavelength at which acceptor emission is measured. Because of these two types of signal contaminations, the ratiometric method (acceptor emission/donor emission) cannot accurately measure the absolute FRET signal, as it doubles the effect of bleed-through emission. Another effort to estimate the dissociation constant *K*_*d*_ by FRET assay was pioneered by Erickson *et al*., who measured quantitative “three-cube” FRET using fluorescent microscopy^2,3^. However, this methodology requires detailed knowledge of optical filter characteristics such as the average molar extinction co-efficiency of donor and acceptor over the bandwidth of FRET cube excitation filter. Although several improvements have been developed, microscopy images are blurred by the optical imaging process from out-focus image planes and adjacent points^4,5^. In addition, an independent estimation of FRET efficiency is needed to characterize protein binding. However, the FRET efficiency is affected by multiple factors, and accurate determination is often challenging. Recent studies of non-imaging-based FRET assay for *K*_*d*_ determinations have focused on the development of quantitative methodologies for steady-state and kinetic parameters of protein interactions or enzymatic reactions by photomultipliers (PMT)-based quantitative FRET assay^6–8^. In one study, CFP-SUMO1 and YFP-Ubc9 recombinant proteins were mixed, and the fluorescent spectra were compared with those from the same concentrations of separate CFP-SUMO1 or YFP-Ubc9 proteins to derive the FRET emission from YFP-Ubc9^6^. The FRET emission intensity was then fitted with YFP-Ubc9 concentration to obtain the maximum FRET emission intensity, which is correlated with maximum bindings of two proteins. The bound YFP-Ubc9 concentration was calculated from the FRET emission with the assumption of a linear relationship. In the second study, the individual and quantified absolute fluorescence signals contributed by each component (i.e., donor, acceptor and FRET at the emission wavelength of acceptor) were determined using correlations of donor and acceptor fluorescence emissions. The absolute FRET signal was correlated with the amount of bound partners, which was then used to derive interaction affinity *K*_*d*_^7^. A similar strategy was applied to Sentrin/SUMO-specific proteases 1 (SENP1) for its *k*_*cat*_/*K*_*M*_ determinations^8^. The results from these quantitative FRET analyses are comparable to or more accurate than traditional biophysical or biochemical approaches, such as the surface plasmon resonance (SPR) or Western blot for estimating binding affinity constants. Furthermore, the FRET method provides free molecular interaction in solution and timely signal detection and therefore results in higher kinetic numbers^6,7,9^. In FRET, fluorescence quenching of a donor is proportional to the energy transferred to its acceptor, while fluorescence quenching is a more general approach than fluorescence emission as many FRET acceptors can be excitable fluorophores or quenching fluorophores. The fluorescence quenching approach was pioneered by Velick, *et al*. for characterizing antibody-hapten binding^10^. This approach was further developed by Liu and Schultz for characterizing binding between macromolecules^11^.

Here we report a more general method of quantitative FRET signal analysis-quantitative donor signal quenching for *K*_*d*_ determinations. New mathematical algorithms of nonlinear regression were developed, and experimental data were generated and analyzed. The estimated *K*_*d*_*K*_*d*_ of SUMO1-Ubc9 interaction is in good agreement in general with those determined by the acceptor emission approach and the surface plasmon resonance (SPR). Our analysis is the first documentation of FRET quenching technique for protein dissociation constant determination. In addition, our method has broader applications regardless whether the acceptor is an emitting fluorophore or a quencher.

## Methods

### DNA constructs, protein expression and purification

Most of the plasmid constructs and protein expression procedures have been described^7^. Briefly, CyPet-SUMO1 and YPet-Ubc9 were cloned into the NheI/NotI sites of pET28(b) vector (Novagen). BL21(DE3) *Escherichia coli* cells were transformed with pET28 vectors encoding CyPet-SUMO1 or YPet-Ubc9. The expression of Poly-his tagged recombinant proteins was induced with 0.1 mM IPTG at 25 °C overnight. The recombinant proteins were then purified by Ni^2+^-NTA agarose beads (QIAGEN) and eluted by buffer containing 20 mM Tris-HCl, pH 7.5, 200 mM NaCl, and 150 mM imidazole. After the proteins were dialyzed in buffer containing 20 mM Tris-HCl, pH 7.5, 50 mM NaCl, and 1 mM DTT, they were concentrated and purified by gel filtration HPLC with Superdex75 10/300 GL column with a HPLC purification system (ÄKTA^TM^ purifier. GE Healthcare). Purity of proteins was confirmed by SDS-PAGE and Coomassie blue staining, and concentrations were determined by Coomassie Plus Protein Assay (Thermo-Fisher).

### Fluorescence measurement of donor quenching

The FRET measurements were as previously described^7^. Briefly, recombinant CyPet-SUMO1 and YPet-Ubc9 proteins were diluted with Tris buffer (20 mM Tris-HCl, pH 7.5, 50 mM NaCl) in a total volume of 100 µL. For each set of measurements, the final concentrations of CyPet-SUMO1 were 0.5, 1.0 and 1.5 µM, respectively. The final concentrations of YPet-Ubc9 were increased from 0 to 4 µM. The fluorescence emission spectrum of each sample was determined using a fluorescence multi-well plate reader FlexstationII^384^ (Molecular Devices, Sunnyvale, CA). The fluorescence emission at 475 nm was measured at the excitation wavelength of 414 nm with a cutoff filter of 455 nm. For all the data points, the final fluorescent signals were obtained by subtracting the fluorescent signals with the background noise of blank well. The experiments were repeated three times and the average value of fluorescence were taken at each specific condition. The decrease of emission intensity at 475 nm (ΔEm_475_) is calculated by subtracting the emission intensity at a specific YPet-Ubc9 concentration by the emission intensity of CyPet-SUMO1 only:

$${{\rm{\Delta }}\text{Em}}_{475}={{\rm{Em}}}_{475([{\rm{YPetUbc}}9]={\rm{X}})}-{{\rm{Em}}}_{475([{\rm{YPetUbc}}9]=0)}$$

### Data processing and *K*_*d*_ determination

After ΔEm_475_ at each specific condition was calculated based on the method described above, a non-linear regression model was used to fit the datasets of ΔEm_475_ and the total concentration of YPet-Ubc9 ([YPetUbc9]_total_) by Prism 5 (GraphPad Software) to derive the value of *K*_*d*_. In the non-linear regression model, the values of [YPetUbc9]_total_ were put into X-series and the intensities of ΔEm_475_, which were determined in triplicate at each [YPetUbc9]_total_ were put into Y-series.

### Statistic Bootstrap Analysis

We denoted the *K*_*d*_ estimator based on the two methods by ${\hat{k}}_{d1}$ and ${\hat{k}}_{d2}$, respectively. Based on the data from our experiments, ${\hat{k}}_{d1}=0.46744$ and ${\hat{k}}_{d2}=0.41146$. We determined whether the difference of the two estimates is caused by the variation of the data and the true *K*_*d*_*K*_*d*_ measured by the two methods are actually the same. This is equivalent to testing *H*_0_: *K*_*d*1_ = *K*_*d*2_ versus *H*_1_: *K*_*d*1_ ≠ *K*_*d*2_, where *K*_*d*1_ and *K*_*d*2_ are the true *K*_*d*_ measured by the two methods, respectively. To test this hypothesis, we examined the distribution of our test statistic ${\hat{k}}_{d1}-{\hat{k}}_{d2}$ under *H*_0_. To find out this distribution, we resort to the bootstrap method. More specifically, we simulated a new set of *y*’s from the two models using the estimated *K*_*d*_ and *n* as the true values plus some random noises which mimic the variation of the original data. Based on the new set of *y*’s, we obtained a new set of *K*_*d*_ estimates, denoted by ${\hat{k}}_{d1}^{\ast 1}$ and ${\hat{k}}_{d2}^{\ast 1}$. We repeated this procedure 10,000 times, and the resulting *K*_*d*_ estimates are ${\hat{k}}_{d1}^{\ast 1},\ldots ,\,{\hat{k}}_{d1}^{\ast 10000},$ and ${\hat{k}}_{d2}^{\ast 1},\ldots ,{\hat{k}}_{d2}^{\ast 10000}.$ The null distribution of ${\hat{k}}_{d1}-{\hat{k}}_{d2}$ then could be approximated by the empirical distribution of $({\hat{k}}_{d1}^{\ast 1}-{\hat{k}}_{d2}^{\ast 1})-({\hat{k}}_{d1}-{\hat{k}}_{d2}),\ldots ,({\hat{k}}_{d1}^{\ast 10000}-{\hat{k}}_{d2}^{\ast 10000})-({\hat{k}}_{d1}-{\hat{k}}_{d2})$. Based on the approximated null distribution of ${\hat{k}}_{d1}-{\hat{k}}_{d2}$, we obtained the *p*-value of the above hypothesis testing problem, which is *p* = 0.19148. Since the *p*-value is larger than 0.05, we do not have sufficient evidence to reject *H*_0_ and conclude that the true *K*_*d*_ measured by the two methods are actually identical.

## Results

### Measurement of *K*_*d*_ at the donor emission wavelength

**Figure 1:**
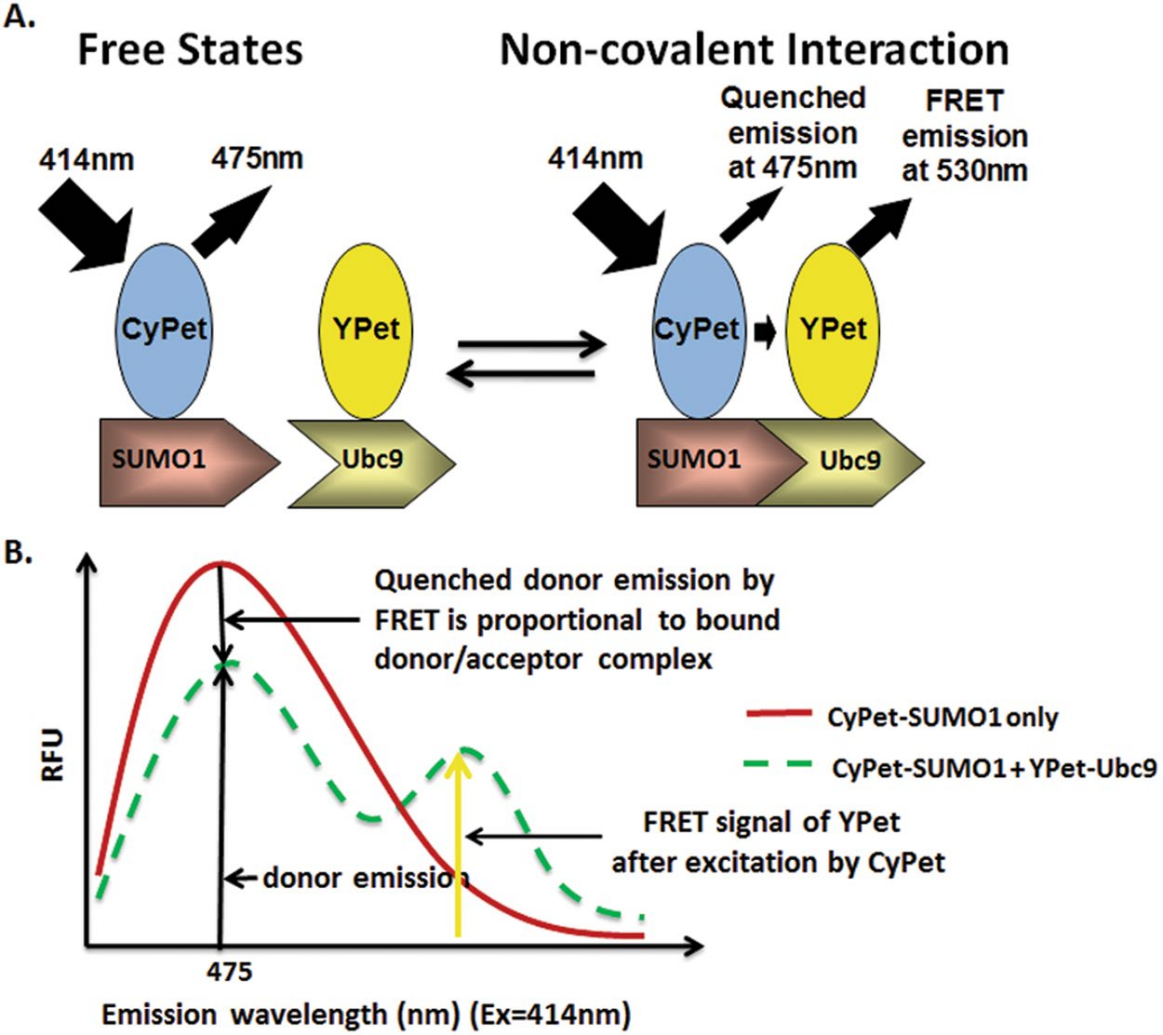
Schematic of the principle of donor quenching for protein interaction affinity determination by FRET assay. (**A**) Diagram of fluorescence emissions of FRET pair, CyPet and YPet, when tagged with interactive partners, SUMO1 and Ubc9, respectively. The fluorescence emission of donor CyPet-SUMO1 decreases when it binds acceptor Ypet-Ubc9. (**B**) Quantitative analysis of fluorescence signals. The decrease of CyPet emission is proportional the concentration of bound donor/acceptor complex.

To determine the dissociation constant, *K*_*d*_ by FRET assay, we chose a high-efficiency FRET pair, CyPet and YPet, to fuse with SUMO1 and Ubc9, respectively (Fig. 1A). CyPet and YPet are fluorescent proteins engineered from CFP and YFP, respectively, with 20-fold greater ratiometric FRET signal than their parental FRET pair^12^.

In our previous development, the interaction affinity *K*_*d*_ was determined from the FRET acceptor emission signal at 530 nm after eliminations of direct emissions of CyPet-SUMO1 and YPet-Ubc9^7^. As the fluorescent signal emitted by acceptor is proportional to the quenched fluorescent signal of donor in the FRET, we reasoned that the decrease in donor signal should also be proportional to the bound complex in the FRET assay, and therefore could be used for *K*_*d*_ determination. However, this is not true in a titration format when dilution also affects the donor emission signal. Therefore, donor concentration needs to be constant in a titration experiment.

Starting with the general law of mass action for protein-protein interaction,

$${\rm{CyPet}}-{\rm{SUMO}}1+{\rm{YPet}}-{\rm{Ubc}}9\leftrightarrow {\rm{CyPet}}-{\rm{SUMO}}1\cdot {\rm{YPet}}-{\rm{Ubc}}9$$

The *K*_*d*_ can be calculated as follows:

1 $${{\rm{K}}}_{{\rm{d}}}=\frac{{[{\rm{CyPetSUMO}}1]}_{{\rm{free}}}{[{\rm{YPetUbc}}9]}_{{\rm{free}}}}{[{\rm{CyPetSUMO}}1\cdot {\rm{YPetUbc}}9]}=\frac{{[{\rm{CyPetSUMO}}1]}_{{\rm{free}}}{[{\rm{YPetUbc}}9]}_{{\rm{free}}}}{{[{\rm{YPetUbc}}9]}_{{\rm{bound}}}}$$

The decrease of emission intensity of CyPet at 475 nm results from the quenching of CyPet-SUMO1 by acceptor YPet-Ubc9 as FRET occurs (Fig. 1B). Because the amount of quenched CyPet-SUMO1 fluorescence is proportional to the amount of bound protein complex, the relationship of emission decrease and concentration of bound protein can be represented by:

2 $${{\rm{\Delta }}\text{Em}}_{475}={\rm{n}}\times {[{\rm{YPetUbc9}}]}_{{\rm{bound}}},$$

where n is a constant related to the FRET efficiency between CyPet-SUMO1 and YPet-Ubc9, [YPetUbc9]_bound_ is the concentration of bound YPet-Ubc9, and ΔEm_475_ is the decrease of emission intensity at 475 nm with an excitation wavelength of 414 nm at each specific concentration of YPet-Ubc9,

3 $${{\rm{\Delta }}\text{Em}}_{475}={{\rm{Em}}}_{475([{\rm{YPetUbc}}9]={\rm{X}})}-{{\rm{Em}}}_{475([{\rm{YPetUbc}}9]=0)}$$

where x is the concentration of YPet-Ubc9 in the FRET assay.

If we set the total concentration of CyPet-SUMO1 to a constant A, the concentration of total YPet-Ubc9 to the variable X, and ΔEm_475_ to the variable Y, we can convert the concentration of bound and free CyPet-SUMO1 or YPet-Ubc9 proteins in Equation 2 to:

4 $${[{\rm{YPetUbc}}9]}_{{\rm{bound}}}=\frac{{\rm{Y}}}{{\rm{n}}}$$

and

5 $${[{\rm{CypetSUMO}}1]}_{{\rm{free}}}={\rm{A}}-\frac{{\rm{Y}}}{{\rm{n}}}$$

and

6 $${[{\rm{YPetUbc}}9]}_{{\rm{free}}}={\rm{X}}-\frac{{\rm{Y}}}{{\rm{n}}}$$

Based on the definition of *K*_*d*_ and Equations 4 to 6, we can derive

7 $${{\rm{K}}}_{{\rm{d}}}=\frac{({\rm{A}}-\frac{{\rm{Y}}}{{\rm{n}}})({\rm{X}}-\frac{{\rm{Y}}}{{\rm{n}}})}{\frac{{\rm{Y}}}{{\rm{n}}}}=\frac{({\rm{nA}}-{\rm{Y}})({\rm{nX}}-{\rm{Y}})}{{\rm{nY}}}$$

After rearranging the above equations, the following equations are obtained:

8 $${{\rm{Y}}}^{2}-{\rm{n}}({\rm{A}}+{\rm{X}}){\rm{Y}}+{{\rm{n}}}^{2}{\rm{AX}}={{\rm{K}}}_{{\rm{d}}}{\rm{nY}}$$

9 $${{\rm{Y}}}^{2}-{\rm{n}}({\rm{A}}+{\rm{X}}+{{\rm{K}}}_{{\rm{d}}}){\rm{Y}}+{{\rm{n}}}^{2}{\rm{AX}}=0$$

10 $${\rm{Y}}=\frac{{\rm{n}}}{2}({\rm{A}}+{\rm{X}}+{{\rm{K}}}_{{\rm{d}}}-\sqrt{{({\rm{A}}+{\rm{X}}+{{\rm{K}}}_{{\rm{d}}})}^{2}-4\text{AX}\,})$$

Therefore, by fitting ΔEm_475_ (Y) and the total YPet-Ubc9 concentration (X) with Equation 10, we can derive the value of *K*_*d*_ and constant n.

### Determination of the quenched donor emission ΔEm_475_

**Figure 2:**
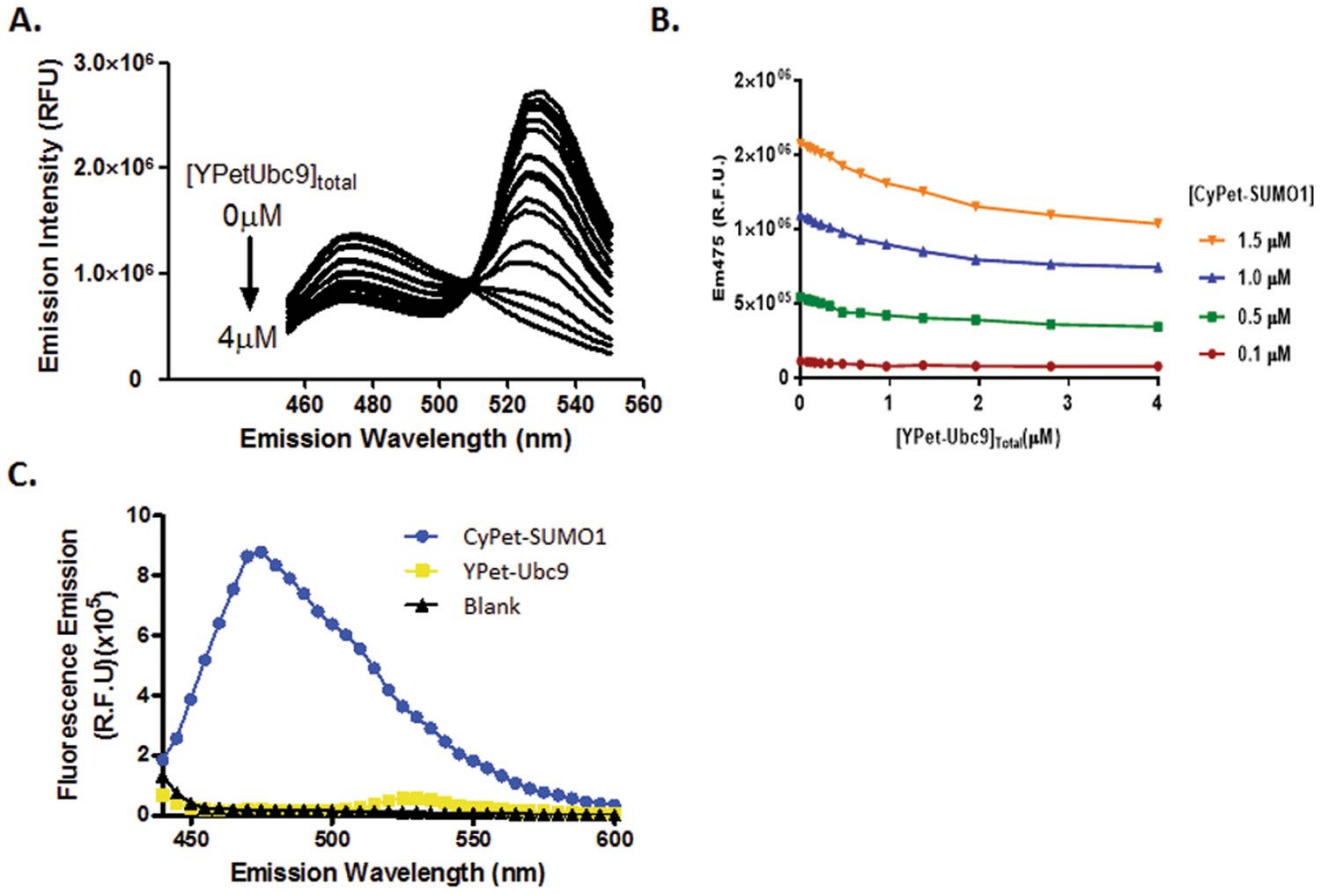
Fluorescence emission from the mixture of CyPet-SUMO1 and YPet-Ubc9. (**A**) Spectrum changes of the mixture of CyPet-SUMO1 and YPet-Ubc9 proteins at increasing amount of acceptor when excited at 414 nm. The CyPet-SUMO1 concentration is fixed at 1.5 μM, and YPet-Ubc9 concentrations range from 0 to 4 μM. (**B**) Emission intensity of CyPet-SUMO1 at 475 nm (Ex = 414 nm) decreases at different concentrations of CyPet-SUMO1 with increasing YPet-Ubc9 concentrations. ● 0.5 μM of CyPet-SUMO1, ■ 1 μM of CyPet-SUMO1, and ▲ 1.5μM of CyPet-SUMO1. (**C**) The emissions of CyPet and YPet when excited at 441 nm.

The *K*_*d*_ can be determined with a series of FRET assays, in which a single constant concentration of CyPet-tagged protein was used together with increasing concentrations of YPet-tagged interactive partner. However, to test the robustness of our approach, we set up the FRET experiments at different concentrations of CyPet-tagged protein. In our effort to determine *K*_*d*_, we fixed the CyPet-SUMO1 concentration at 0.1, 0.5, 1.0, or 1.5 μM and increased the concentration of YPet-Ubc9 from 0 to 4 μM in a total volume of 100 μl^13^. The fluorescence emission spectra of the mixtures were then determined with a FlexstationII^384^ at an excitation wavelength of 414 ± 4 nm (Fig. 2A). As the concentration of YPet-Ubc9 was increased, the emission intensity at 475 ± 4 nm gradually decreased, indicating that CyPet-SUMO1 molecules were bound to YPet-Ubc9 and quenched by donor YPet-Ubc9 in the FRET assay. The range of acceptor signal increase was larger than that of donor signal decrease (Fig. 2A).

The absolute quenched signal of CyPet-SUMO1 was obtained by Equation 3. At each CyPet concentration, the magnitude of quenching was obtained by subtracting the total fluorescence emission at 475 nm in the absence of YPet-Ubc9 from the remaining fluorescence emission at 475 nm in the presence of YPet-Ubc9 at different concentrations of YPet-Ubc9. The quenched signal of CyPet-SUMO1 shows a gradually decrease with increasing concentrations of YPet-Ubc9 (Fig. 2B). These data demonstrate that the degree of quenching of donor fluorescence increases with increased concentration of YPet-Ubc9 and it can be used to determine bound protein of protein interactions. In addition, because the YPet does not give emission at 475 nm when excited at 414 nm, this simplifies our analysis with only the emission of CyPet at 475 nm considered (Fig. 2C).

### Determination of interaction affinity *K*_*d*_ by quenched donor emission

**Figure 3:**
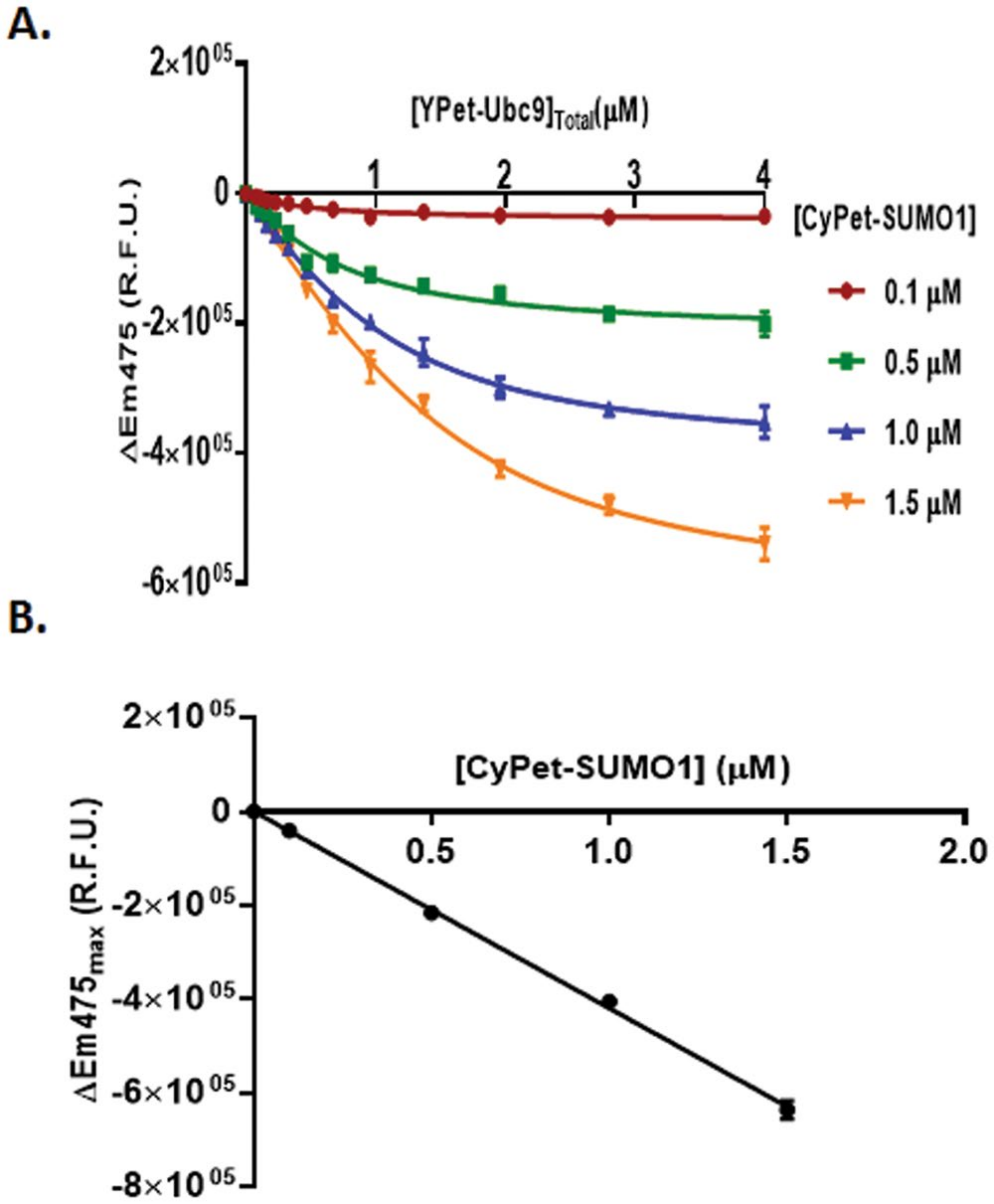
Curve fitting for *K*_*d*_ determination. (**A**) Decreased emission of Em_475_ was calculated by subtracting the emission intensity of CyPet-SUMO1 in the presence of YPet-Ubc9 from the emission in the absence of YPet-Ubc9. The value of decrease was then fitted with total YPet-Ubc9 concentration to derive the value of *K*_*d*_ according the formula Y = n/2 (A + X + *K*_*d*_ − √((A + X + *K*_*d*_)^2−4AX)) (see text for details). (**B**) Linearity of maximum quenching signal of CyPet-SUMO1 by Ypet-Ubc9 at different concentrations was determined.

To determine the *K*_*d*_ and constant n simultaneously from the quenched FRET signals, we applied Equation 10 to ΔEm_475_ determined at each set of experiments with different [YPetUbc9]_total_, and fit the data using nonlinear regression. To apply the Equation 10, we determined ΔEm_475_ at each set of experiments with different [YPetUbc9]_total_, and then modeled the data into the equation by a least-square fitting. Four regression plots corresponding to the concentrations of CyPet-SUMO1 in our experiments (0.1, 0.5, 1.0, and 1.5 μM) were generated (Fig. 3). Nonlinear regression fitted the data reasonably well (R^2^ from 0.95 to 0.99).

From the nonlinear regression of Equation 10, the *K*_*d*_ value can be determined from two parameter non-linear regression (n and *K*_*d*_) because Y(CyPet quenched signal) and X(YPet-Ubc9 concentration) were known from experiments. The *K*_*d*_ values for these four independent experiments corresponding to different CyPet-SUMO1 concentrations were 0.40 ± 0.05, 0.45 ± 0.06, 0.46 ± 0.05, and 0.51 ± 0.07 μM, respectively (see Table 1). These *K*_*d*_ values are very similar to each other and also very close to previous results with acceptor excitation emission for *K*_*d*_ determination and are also very close to the results from other technologies(see Table 1)^6,7^ (see below).

**Table 1:** Summary of fitting results for *K*_*d*_ determination.

	Donor Quenching				Acceptor Emission				Global Optimization	
[CyPet-SUMO1](μM)	0.1	0.5	1	1.5	0.1	0.5	1	1.5	Donor Quenching	Acceptor Emission
*K*_*d*_ (mM)	0.40 ± 0.05	0.45 ± 0.06	0.46 ± 0.05	0.51 ± 0.07	0.36 ± 0.02	0.36 ± 0.01	0.41 ± 0.03	0.42 ± 0.06	0.47 ± 0.03	0.41 ± 0.02

By nonlinear regression, the constant n was also determined in each concentration of CyPet-SUMO1. The n numbers at each concentrations of CyPet-SUMO1 obtained from the non-linear regression were as −4.07 ± 0.15 × 10^5^, −4.32 ± 0.15 × 10^5^, −4.06 ± 0.10 × 10^5^ and −4.24 ± 0.13 × 10^5^, respectively. From its definition, n is a constant that converts the bound acceptor partner to the quenched signal of the donor (Equation 2). The slight fluctuation of n from these four sets of experiments likely suggest that the method is not equally sensitive at different dynamic ranges of readouts, in agreement with the results of different slopes at different concentrations.

### Comparing affinity *K*_*d*_ determinations by donor quenching with FRET emission from acceptor

We previously developed a FRET-based method to determine *K*_*d*_ by acceptor excited emission^7,13^. That approach derives the FRET emission of acceptor from the whole spectrum by subtracting individual contributions of donor and acceptor. The *K*_*d*_ was then derived from the non-liner regression plot of the FRET emission plotted against the total concentration of acceptor.

**Figure 4:**
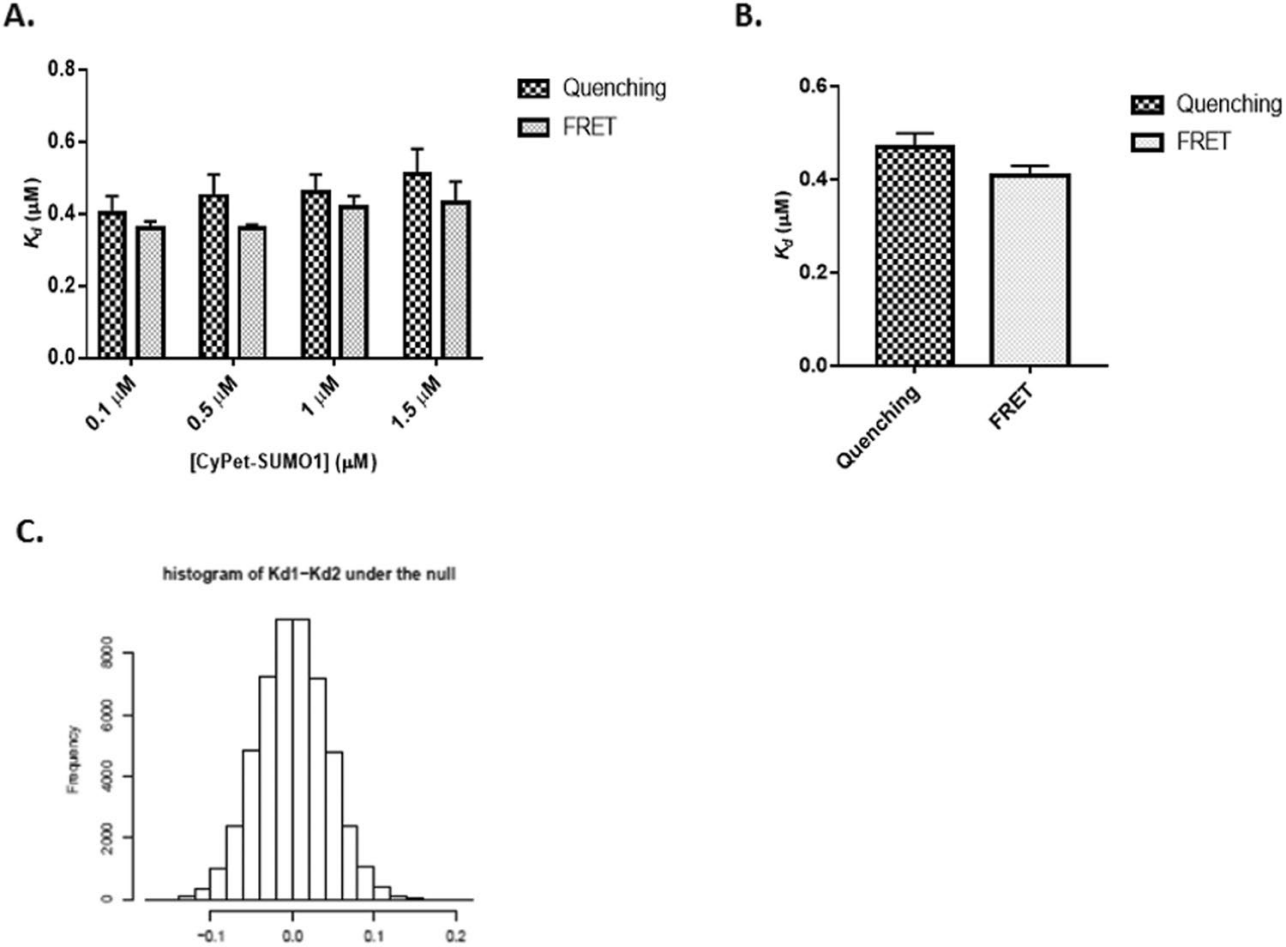
Comparison of *K*_*d*_ values obtained from two approaches by the quenched donor emission or acceptor emission. (**A**) *K*_*d*_ values determined by either FRET quenching or emission at different concentrations of CyPet-SUMO1 were compared. (**B**) *K*_*d*_ values after global optimization of either FRET quenching or emission datasets. (**C**) Bootstrap analysis of both *K*_*d*_ values revealed no significant difference.

To directly compare the sensitivity and accuracy of our newly developed donor quenching method and the FRET emission method, we also used FRET emission method to analyze the same data from the experiments at four different conditions in which [CyPet-SUMO1] is set to 0.1, 0.5, 1.0, or 1.5 μM, respectively and compared the values of *K*_*d*_ with those obtained using the donor quenching method. The *K*_*d*_ values obtained from the method of acceptor FRET emission were 0.36 ± 0.02, 0.36 ± 0.01, 0.41 ± 0.03, and 0.42 ± 0.06 μM at the same concentrations of CyPet, respectively (Fig. 4A). The data were similar across different concentration sets of experiment. To minimize the effect of variations, we performed global optimization for the *K*_*d*_ values from the two approaches, and the resulting *K*_*d*_ values from fluorescence quenching and emission are 0.47 ± 0.03 and 0.41 ± 0.02, respectively (Fig. 4B). These results suggest that the two methods give similar and consistent *K*_*d*_ measurements. We denote the *K*_*d*_ estimators using the two methods by ${\hat{K}}_{d1}$ and ${\hat{K}}_{d2}$, respectively. Based on the data from our experiments, ${\hat{K}}_{d1}=0.47$ and ${\hat{K}}_{d2}=0.41$. We next need to find out whether the difference of the two estimates is caused by the variation of the data and the true *K*_*d*_ measured by the two methods are actually the same. This is equivalent to testing ${H}_{0}:\,{K}_{d1}={K}_{d2}$ versus ${H}_{1}:\,{K}_{d1}\ne {K}_{d2}$, where *K*_*d1*_ and *K*_*d2*_ are the true *K*_*d*_ measured by the two methods, respectively. To test this hypothesis, we need to know the distribution of our test statistic ${\hat{K}}_{d1}-{\hat{K}}_{d2}$ under *H*_0_. To find out this distribution, we resort to the bootstrap method. More specifically, we simulate a new set of *y*’s from the two models using the estimated *K*_*d*_ and *n* as the true values plus some random noises which mimic the variation of the original data. Based on the new set of *y*’s, we obtain a new set of *K*_*d*_ estimates, denoted by ${\hat{K}}_{d1}^{\ast 1}$ and ${\hat{K}}_{d2}^{\ast 1}$. We repeat this procedure 10000 times, and the resulting *K*_*d*_ estimates are ${\hat{K}}_{d1}^{\ast 1},\ldots ,\,{\hat{K}}_{d1}^{\ast 10000},$ and ${\hat{K}}_{d2}^{\ast 1},\ldots ,{\hat{K}}_{d2}^{\ast 10000}.$ The null distribution of ${\hat{K}}_{d1}-{\hat{K}}_{d2}$ then can be approximated by the empirical distribution of $({\hat{K}}_{d1}^{\ast 1}-{\hat{K}}_{d2}^{\ast 1})-({\hat{K}}_{d1}-{\hat{K}}_{d2}),\ldots ,({\hat{K}}_{d1}^{\ast 10000}-{\hat{K}}_{d2}^{\ast 10000})-({\hat{K}}_{d1}-{\hat{K}}_{d2})$. Figure 4 shows the approximated null distribution of ${\hat{K}}_{d1}-{\hat{K}}_{d2}$ if the two *K*_*d*_ are indeed equal using the bootstrap method. The observed ${\hat{K}}_{d1}-{\hat{K}}_{d2}$ from our experiment is 0.06 = 0.47 − 0.41. Based on the approximated null distribution of ${\hat{K}}_{d1}-{\hat{K}}_{d2}$, we obtain the *p*-value of the above hypothesis testing problem, which is *p* = 0.19148. Since the *p*-value is larger than 0.05, we do not have sufficient evidence to reject *H*_0_ and conclude that the true *K*_*d*_ measured by the two methods are actually the same. Our *K*_*d*_ estimates are also very close to the *K*_*d*_ values from surface plasma resonance (BIACORE)(0.35 μM)^7^ and and isothermal titration calorimetry (0.25 μM)^14^, indicating a good agreement with others results.

## Discussion

We have successfully developed a new mathematical algorithm, which allows us to derive *K*_*d*_ values from the FRET assay. This approach complements the previous approach utilizing acceptor fluorescence emission of FRET signal to determine the bound partner concentration and *K*_*d*_, with an advantage of broader applications^6,15^. Like the previous approach, the new method offers an accurate and simplified *K*_*d*_ determination in a one-step procedure. Because YPet does not have an emission at 475 nm when excited at 414 nm, it does not contribute to the emission at 475 nm (Fig. 2C). The FRET signal can be derived from the total emission at 475 nm in the absence of acceptor by subtracting the emission at donor wavelength in the presence of acceptor. The quenched FRET signal is then converted to the concentration of bound partners, which is used to calculate affinity, *K*_*d*_. The very similar *K*_*d*_ values generated from different concentrations and ratios of CyPet-SUMO1 and YPet-Ubc9 (from 0.1 to 1.5 μM of CyPet-SUMO1 and from a ratio of the binding partners of 4 to 40 fold) also demonstrate that the FRET-based *K*_*d*_ measurement approach is reliable and can provide consistent estimates of *K*_*d*_.

The FRET-based *K*_*d*_ measurements, either from donor quenching-based or acceptor excitation-based methods, can provide several advantages over other current *K*_*d*_ measurement methods, such as radio-labeled ligand binding assay, such as SPR or ITC. First, fluorescent protein-tagged interaction partners provide FRET measurement in a solution phase, under conditions more physiological where the *K*_*d*_ is most likely to be close to the affinity of protein interaction in living cells, whereas other methods, such as SPR, require that the conjugations take place on a chip surface, which could interfere with the free interactions of proteins. Second, FRET-based method is very environmental friendly, and protein labeling method is universal. Other methods, such as radio-labeled method, need radioisotopes and special protection tools, and the labeling methodology is very tedious and varies depending on molecules. Third, FRET-based *K*_*d*_ measurement can potentially measure proteins interactions in the presence of other molecules, such as contaminated proteins, while other methods, such as ITC, require very pure proteins. Fourth, FRET-based *K*_*d*_ measurement only requires general fluorescence readers or fluorescence microscopes that are widely available. The Other approaches for *K*_*d*_ determination, such as SPR or ITC, require special instrumentation^16,17^. Finally, our approach provides a general platform for *K*_*d*_ measurements as long as the two interactive partners can be labeled by a FRET pair, such as protein-protein, protein–small molecule or small molecule–small molecule interactions. The mathematical algorithm and experimental procedure of *K*_*d*_ measurement by FRET technology can be generally applied to these cases.

These two approaches, namely donor quenching and acceptor emission, can be used in FRET-based methods for protein affinity determination. Compared to the method using increase in the acceptor emission, the method using quenched donor emission has more general applications because the acceptor can be excited fluorophores or quenchers. Different mathematical algorithms have been developed for these two approaches. The sensitivity and accuracy of FRET assays are dependent on the instrumentation and quantum yield of each partner of a FRET pair. Recently, extensive efforts have been made to develop new fluorescent proteins or small molecules for FRET assays^18–20^. Our methodologies of quantitative FRET assays for protein affinity and enzyme kinetics determination will provide powerful quantitative tools for future applications.

## Data Availability

The materials and protocols are available to public.
